# Immunity elicited by natural infection or Ad26.COV2.S vaccination protects hamsters against SARS-CoV-2 variants of concern

**DOI:** 10.1126/scitranslmed.abj3789

**Published:** 2021-09-07

**Authors:** Lisa H. Tostanoski, Jingyou Yu, Noe B. Mercado, Katherine McMahan, Catherine Jacob-Dolan, Amanda J. Martinot, Cesar Piedra-Mora, Tochi Anioke, Aiquan Chang, Victoria M. Giffin, David L. Hope, Huahua Wan, Esther A. Bondzie, Shant H. Mahrokhian, Linda M. Wrijil, Katherine Bauer, Laurent Pessaint, Maciel Porto, Joseph Piegols, Andrew Faudree, Brittany Spence, Swagata Kar, Fatima Amanat, Florian Krammer, Hanne Andersen, Mark G. Lewis, Frank Wegmann, Roland Zahn, Hanneke Schuitemaker, Dan H. Barouch

**Affiliations:** 1 Center for Virology and Vaccine Research, Beth Israel Deaconess Medical Center, Harvard Medical School, Boston, MA 02115, USA.; 2 Harvard Medical School, Boston, MA 02115, USA.; 3 Department of Biomedical Sciences, Section of Pathology, Cummings School of Veterinary Medicine, Tufts University, North Grafton, MA 01536, USA.; 4 BIOQUAL Inc., Rockville, MD 20850, USA.; 5 Icahn School of Medicine at Mount Sinai, New York, NY 10029, USA.; 6 Janssen Vaccines & Prevention BV, Leiden, 2333 CN, Netherlands.; 7 Ragon Institute of MGH, MIT and Harvard, Cambridge, MA 02139, USA.; 8 Massachusetts Consortium on Pathogen Readiness, Boston, MA 02115, USA.

## Abstract

Severe acute respiratory syndrome coronavirus 2 (SARS-CoV-2) variants of concern have emerged and may pose a threat to both the efficacy of vaccines based on the original WA1/2020 strain and the natural immunity induced by infection with earlier SARS-CoV-2 variants. We investigated how mutations in the spike protein of circulating SARS-CoV-2 variants, which have been shown to partially evade neutralizing antibodies, affect natural and vaccine-induced immunity. We adapted a Syrian hamster model of moderate to severe clinical disease for two variant strains of SARS-CoV-2: B.1.1.7 (alpha variant) and B.1.351 (beta variant). We then assessed the protective efficacy conferred by either natural immunity from WA1/2020 infection or by vaccination with a single dose of the adenovirus serotype 26 vaccine, Ad26.COV2.S. Primary infection with the WA1/2020 strain provided potent protection against weight loss and viral replication in lungs after rechallenge with WA1/2020, B.1.1.7, or B.1.351. Ad26.COV2.S induced cross-reactive binding and neutralizing antibodies that were reduced against the B.1.351 strain compared with WA1/2020 but nevertheless still provided robust protection against B.1.351 challenge, as measured by weight loss and pathology scoring in the lungs. Together, these data support hamsters as a preclinical model to study protection against emerging variants of SARS-CoV-2 conferred by prior infection or vaccination.

## Resisting rechallenge

Prior infection and vaccination have been shown to elicit immunity against severe acute respiratory syndrome coronavirus 2 (SARS-CoV-2). However, the extent to which prior infection or vaccination protects against variants of concern is unclear. In this pair of manuscripts, Chandrashekar *et al*. and Tostanoski *et al*. evaluated the ability of infection with the WA1/2020 strain to protect rhesus macaques and hamsters against secondary challenge with other strains of SARS-CoV-2. In both cases, prior infection elicited immunity that could protect against infection with heterologous strains. In addition, Tostanoski *et al*. showed that vaccination with Ad.26.COV2.S protected hamsters against heterologous SARS-CoV-2 challenge. Together, these data show that prior infection and vaccination are protective against infection with SARS-CoV-2 variants.

**Figure:**
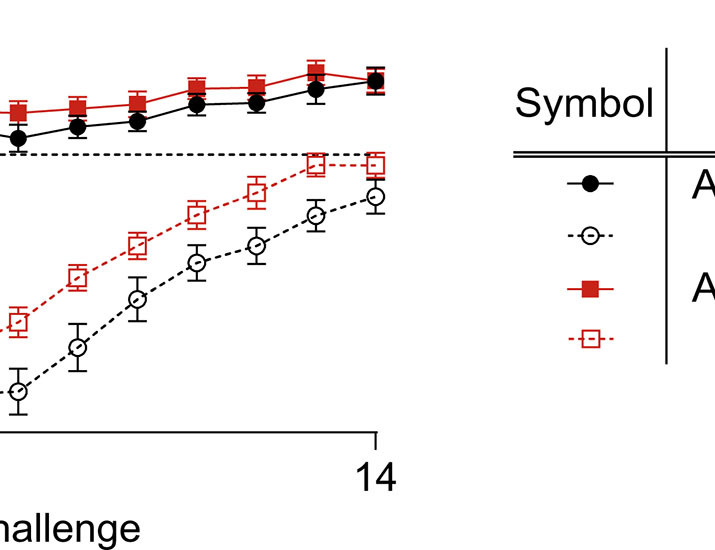

## Teaser

Immunity from prior infection or Ad26.COV2.S vaccination protected hamsters from challenge with a heterologous strain of SARS-CoV-2 5 weeks later.

## INTRODUCTION

The emergence and rapid spread of severe acute respiratory syndrome coronavirus 2 (SARS-CoV-2) variants have raised important questions about the potential impact on both natural and vaccine-elicited immunity. The B.1.1.7 or alpha variant, initially identified in the United Kingdom, has demonstrated enhanced transmissibility (*1*, *2*), whereas the B.1.351 or beta variant, initially identified in South Africa, exhibits partial evasion of antibody responses (*2*–*6*). Understanding how the mutations present in the spike proteins of these variants affect humoral immune responses, viral loads, and clinical disease outcomes could have substantial implications for natural and vaccine-mediated control of the coronavirus disease 2019 (COVID-19) pandemic. Limited clinical data are available in the context of SARS-CoV-2 variants on the susceptibility of previously exposed individuals to reinfection or impact on disease severity. We sought to explore these questions in hamsters, which is a model for moderate to severe SARS-CoV-2 clinical disease (*7*–*10*). We reasoned that this approach might provide insight into the potential for immunologic control of SARS-CoV-2 variants after primary infection with the WA1/2020 strain.

A key related question is how variants may affect vaccine-elicited immunity. We previously investigated the protective efficacy of Ad26.COV2.S in nonhuman primate and hamster challenge models (*9*, *11*–*13*). Recent data in nonhuman primates reveal that neutralizing antibody responses are partially attenuated against a B.1.351 pseudotype virus (*12*). Furthermore, in the phase 3 efficacy trial of Ad26.COV2.S, protection from severe COVID-19 was observed, with efficacy of 86, 88, and 82% in the United States, Brazil, and South Africa, respectively (*14*). Critically, a substantial proportion of the viruses sequenced in the trial were variant strains, with 69% of sequences corresponding to the P.2 or zeta variant in Brazil and 95% of sequences corresponding to the B.1.351 variant in South Africa. These data and the emergence of new circulating strains globally support the importance of developing animal models to study the impact of variants on vaccine-mediated protection.

## RESULTS

### SARS-CoV-2 variant strains induce disease in a hamster challenge model

**Fig. 1.**
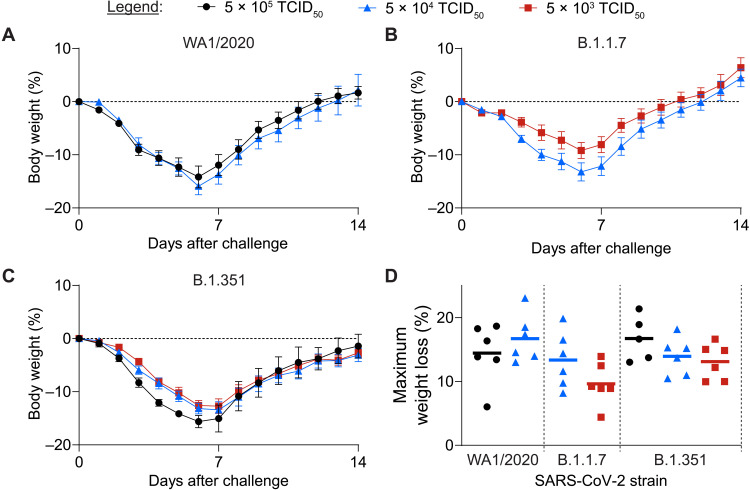
SARS-CoV-2 challenge with variant strain viruses drives clinical disease in hamsters. Groups of Syrian golden hamsters were challenged intranasally with WA1/2020, B.1.1.7, or B.1.351 variant challenge virus in 100 μl of volume using the indicated dose of each challenge stock. After challenge, hamsters were monitored for 14 days for clinical signs of disease. (**A** to **C**) Average relative body weight of each group is displayed as means ± SEM for hamsters challenged with WA1/2020 (A), B.1.1.7 (B), or B.1.351 (C) variant strain challenge stock (*n* = 5 to 6 hamsters per group). (**D**) A comparison of the peak weight loss across variant virus strains and challenge doses is shown. Horizontal lines in (D) indicate the group means.

We expanded stocks of WA1/2020, B.1.1.7, and B.1.351 SARS-CoV-2 variants through in vitro passage in either VeroE6 or Calu-3 cells. Deep sequencing confirmed the expected corresponding sequences for each challenge stock and revealed no unexpected or additional mutations; in particular, no mutations or deletions with >2.5% frequency in the Spike furin cleavage site were detected [National Center for Biotechnology Information (NCBI) Sequence Read Archive (SRA) accession numbers SRR 14313077 and 14313078]. We inoculated groups of Syrian hamsters by the intranasal route with each variant at 5 × 10^5^, 5 × 10^4^, and 5 × 10^3^ median tissue culture infectious dose (TCID_50_) of these stocks. Challenge with the WA1/2020 variant led to severe (greater than 15%) weight loss by day 6, consistent with our prior published data (Fig. 1A and fig. S1) (*9*). Challenge with the B.1.1.7 (Fig. 1B and fig. S1) and B.1.351 (Fig. 1C and fig. S1) variants led to comparable kinetics and extent of weight loss, with no statistically significant differences observed in peak weight loss across variants or challenge doses (*P* > 0.08; Fig. 1D).

### Natural immunity confers protection against rechallenge with heterologous variants

**Fig. 2.**
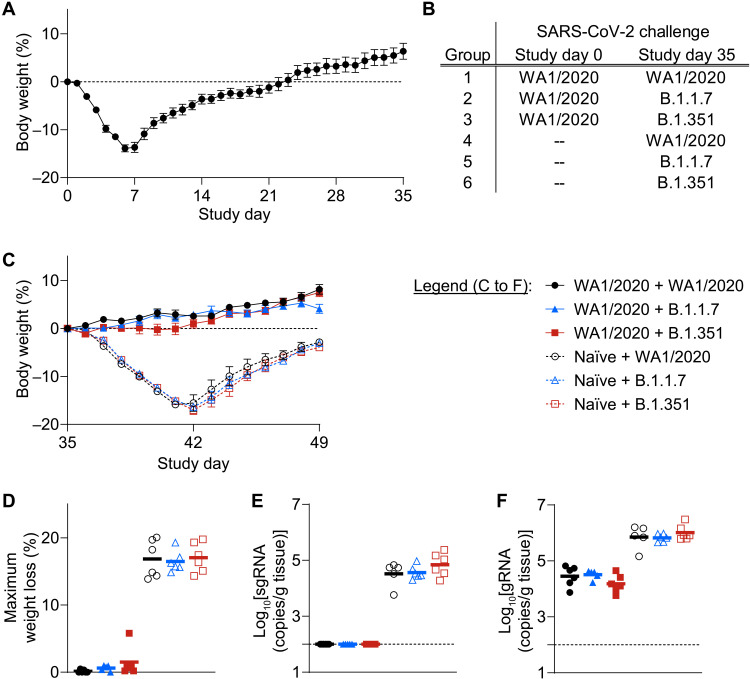
Infection with WA1/2020 SARS-CoV-2 provides natural protection from homologous or heterologous strain rechallenge-induced weight loss. (**A**) Hamsters (*n* = 18) were challenged on day 0 with 5 × 10^4^ TCID_50_ (in 100 μl) of WA1/2020 SARS-CoV-2 challenge stock by the intranasal route and monitored for 35 days after challenge. Average relative body weight after primary challenge displayed as means ± SEM. (**B**) At week 5, hamsters were divided into three groups and rechallenged with 5 × 10^4^ TCID_50_ of WA1/2020 (group 1, *n* = 6), B.1.1.7 (group 2, *n* = 5), or B.1.351 (group 3, *n* = 6) SARS-CoV-2. In addition, three groups of naïve hamsters (*n* = 6 per group) were challenged on study day 35 with the matched strains and doses (groups 4 to 6). (**C**) To allow comparison of weight loss after SARS-CoV-2 challenge or re-challenge on day 35, for the remainder of the study, hamster weights were normalized to the weight on study day 35. Average relative weight after primary challenge (open symbols) or rechallenge (closed symbols) is indicated as means ± SEM. (**D**) Peak weight loss over the span of study days 35 to 49 is shown. Maximum weight loss of each hamster is displayed, with group means indicated by horizontal lines. (**E** and **F**) At the terminal time point, day 49, hamsters were euthanized, and lung tissue was collected to quantify sgRNA (E) and gRNA (F) viral RNA. Log-transformed viral loads in individual hamsters, normalized per gram of lung tissue, are displayed. Horizontal lines indicate group means.

We next explored the potential of natural immunity from a primary WA1/2020 infection to protect against rechallenge with either homologous or heterologous variants. Eighteen hamsters were infected with 5 × 10^4^ TCID_50_ WA1/2020 strain and monitored for 5 weeks after challenge. All hamsters exhibited substantial body weight loss (Fig. 2A), which peaked about 1 week after challenge and was followed by a gradual recovery. At week 5, hamsters were divided into three groups with similar body weight loss (fig. S2) and rechallenged with 5 × 10^4^ TCID_50_ of WA1/2020, B.1.1.7, or B.1.351 SARS-CoV-2 (Fig. 2B).

At the time of rechallenge (week 5 post initial challenge), three additional groups of naïve, age-matched hamsters were also challenged as internal positive controls. Severe weight loss was observed in these naïve animals with all three viruses, similar to the results of the previous experiment (Fig. 2C, open symbols, and fig. S3). Mean maximum weight loss of 16.9, 16.5, and 17.0% was observed after primary challenge with WA1/2020, B.1.1.7, and B.1.351, respectively (Fig. 2D). In contrast, hamsters that had recovered from WA1/2020 infection and were rechallenged maintained body weight with minimal signs of disease for all three viruses (Fig. 2C, closed symbols, and fig. S3). Mean maximum weight loss of 0.1, 0.6, and 1.5% was observed in groups rechallenged with WA1/2020, B.1.1.7, and B.1.351 (Fig. 2D).

At week 7, lung tissue was collected from hamsters to quantify replicating virus in respiratory tract tissue by subgenomic RNA (sgRNA) assays (*15*, *16*). In groups of hamsters that received a primary challenge, persistent sgRNA was detected, with mean values of 3.3 × 10^4^, 3.7 × 10^4^, and 7.0 × 10^4^ copies/g lung tissue, respectively, after WA1/2020, B.1.1.7, and B.1.351 challenge (Fig. 2E). In contrast, no sgRNA was detected in lungs from hamsters that were rechallenged, irrespective of the rechallenge strain (Fig. 2E). A similar pattern was observed in assays to detect genomic RNA (gRNA) (Fig. 2F), with reduced copies per gram of lung tissue after rechallenge, irrespective of variant strain.

Before challenge or rechallenge at week 5, binding antibodies to the variant receptor binding domain (RBD) and spike (S) proteins were quantified by enzyme-linked immunosorbent assay (ELISA) and electrochemiluminescence assays (ECLAs), and neutralizing antibody responses were quantified using pseudovirus neutralization assays. In hamsters that received a primary challenge with WA1/2020, high WA1/2020 and B.1.1.7 binding responses were observed, with geometric mean ELISA titers of 2.0 × 10^4^ and 1.3 × 10^4^, respectively. B.1.351 titers, on average, were about sixfold lower by ELISA, with a geometric mean value of 3.0 × 10^3^ (fig. S4, A to D). After rechallenge, an increase in titers was observed in all rechallenge groups; hamsters that received a primary challenge with each variant also exhibited detectable binding and neutralizing antibody responses (fig. S4, E to H). Together, these data suggest that primary exposure to WA1/2020 SARS-CoV-2 provided natural immunity that restricted weight loss and viral replication in lungs after rechallenge with either the homologous WA1/2020 strain or the B.1.1.7 and B.1.351 variants. Furthermore, this robust protection was observed against B.1.351 rechallenge, despite observed reduced antibody titers after WA1/2020 infection.

### Ad26.COV2.S-elicited humoral immune responses confer protection against variant strains of SARS-CoV-2

**Fig. 3.**
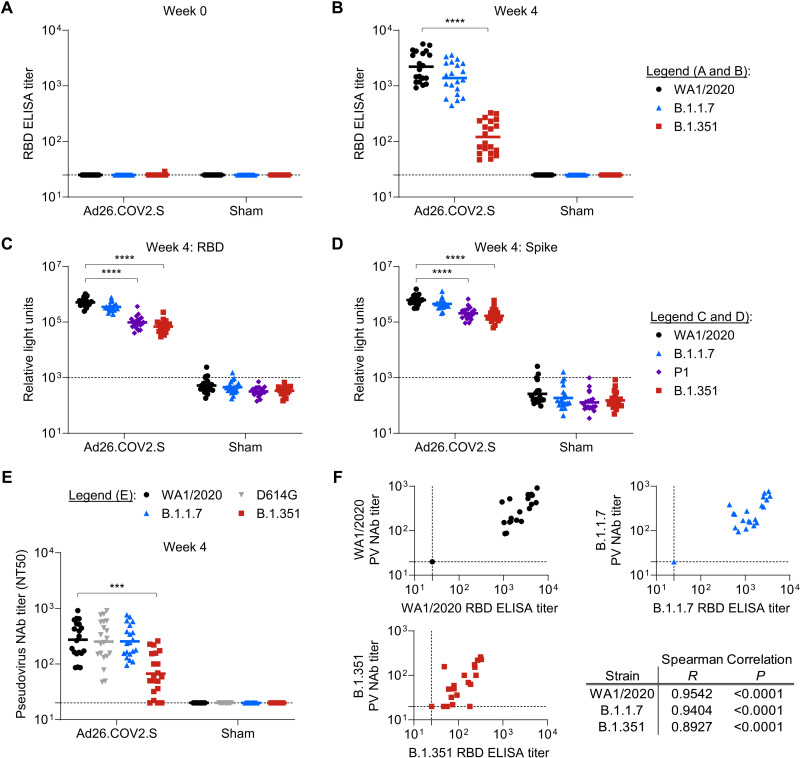
Characterization of Ad26.COV2.S-elicted variant RBD-specific binding and neutralizing antibodies. Groups of hamsters (*n* = 10 per group) were immunized with 10^10^ viral particles of Ad26.COV2.S or a sham control (buffer only), and prechallenge humoral immune responses were quantified in serum. (**A** and **B**) Binding antibodies to WA1/2020, B.1.1.7, and B.1.351 RBD proteins were quantified by ELISA at weeks 0 (A) and 4 (B) after immunization. Horizontal lines indicate group geometric mean titer. (**C** and **D**) ECLAs were used to quantify binding to indicated variant strain RBD (C) and spike proteins (D). Horizontal lines indicate group geometric mean titer. (**E**) Pseudovirus (PV) neutralization assays were used to quantify neutralizing antibody (NAb) titers against pseudoviruses expressing SARS-CoV-2 spike protein specific for the WA1/2020, WA1/2020-D614G, B.1.1.7, or B.1.351 variant at week 4 after immunization. Data displayed are the NT50 values for each hamster; horizontal lines indicate group geometric mean titers. (**F**) Correlation analyses of binding and neutralizing titers for the indicated SARS-CoV-2 variant strains. Summary Spearman correlation results are displayed in the table. Statistics in (A) to (E) indicate the results of Kruskal-Wallis tests with Dunn’s multiple comparisons test (***P < 0.001; ****P < 0.0001). Horizontal dashed lines in (A) to (E) indicate the assay limits of quantitation.

An important question in the field is whether current vaccine designs, which are largely based on the WA1/2020 spike sequence, can elicit robust immune responses that protect against challenge with variants of concern. Thus, we sought to characterize humoral immune responses after Ad26.COV2.S immunization and efficacy against the SARS-CoV-2 variant of concern B.1.351. We immunized 20 hamsters with 10^10^ viral particles of Ad26.COV2.S and 20 hamsters with sham vaccine. We characterized Ad26.COV2.S-elicited immune responses by assessing binding and neutralizing antibody titers. At baseline, hamsters exhibited minimal binding titers to WA1/2020, B.1.1.7, or B.1.351 RBD proteins (Fig. 3A). Four weeks after Ad26.COV2.S immunization, binding titers were observed against the WA1/2020 RBD with geometric mean titers of 2208 (Fig. 3B). Similar binding titers were observed in B.1.1.7 RBD–specific binding assays (geometric mean titer: 1384), whereas reduced titers were observed against the B.1.351 RBD (geometric mean titer: 121), as expected.

We next conducted ECLAs to quantify binding to both variant RBD proteins and variant full-length spike proteins. Similar to ELISA data, we observed the highest signal against WA1/2020 RBD in Ad26.COV2.S-immunized hamsters, with a geometric mean relative light unit (RLU) signal of 5.1 × 10^5^ (Fig. 3C). Similar binding was observed against the B.1.1.7 RBD (3.5 × 10^5^), whereas reduced RLU values were detected against the P.1 (9.8 × 10^4^) and B.1.351 (6.9 × 10^4^) variant RBDs. In parallel ECLAs to full-length spike protein, similar trends were observed, with geometric mean titers of 6.2 × 10^5^, 4.5 × 10^5^, 2.1 × 10^5^, and 1.7 × 10^5^ against WA1/2020, B.1.1.7, P.1, and B.1.351 variants, respectively (Fig. 3D). These data reveal a larger impact of the variant spike mutations on binding to the B.1.351 RBD than the B.1.351 full-length spike.

To characterize antibody function, in vitro variant pseudovirus neutralization assays were also conducted at week 4 after vaccination. Similar to binding assays, immunization with Ad26.COV2.S elicited robust 50% neutralization titers (NT50) against pseudovirus expressing the WA1/2020 SARS-CoV-2 Spike as previously described (Fig. 3E) (*9*, *11*, *17*, *18*). Comparable geometric mean NT50 values were observed in Ad26.COV2.S-immunized hamsters for WA1/2020 (274), WA1/2020-D614G (253), and B.1.1.7 (255) variant pseudoviruses. Reduced neutralizing titers were observed against the B.1.351 variant pseudovirus, with geometric mean titers of 67, representing a 4.1-fold reduction compared with the original WA1/2020 titer. NT50 titers against B.1.351 were lower after vaccination than natural infection. Correlation analyses of binding and neutralizing were performed for each variant strain (Fig. 3F). For WA1/2020, B.1.1.7, and B.1.351 strains, correlations (*P* < 0.0001, Spearman analyses) were observed with strong positive associations reflected in *R* values of 0.9542, 0.9494, and 0.8927, respectively.

### Protective efficacy of Ad26.COV2.S against WA1/2020 and B.1.351

**Fig. 4.**
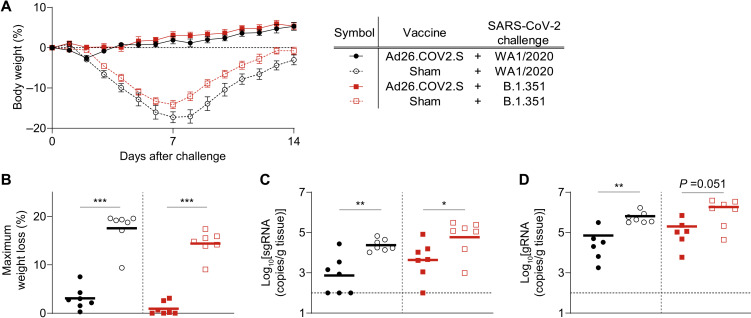
Ad26.COV2.S protects hamsters from WA1/2020 or B.1.351 challenge–induced weight loss. Groups of hamsters (*n* = 10 per group) were vaccinated with either 10^10^ viral particles of Ad26.CoV2.S or sham and then challenged with 5 × 10^4^ TCID_50_ of either WA1/2020 or B.1.351 SARS-CoV-2 by the intranasal route at week 5. (**A**) Average relative body weight of each group is displayed as means ± SEM. (**B**) A comparison of the peak weight loss is shown across experimental groups with horizontal lines indicating the group means. (**C** and **D**) Hamsters were euthanized at day 14 after challenge to quantify sgRNA (C) and gRNA (D) in lung tissue. Horizontal lines indicate group means of log-transformed viral load data. Statistics shown are the results of Mann-Whitney tests comparing Ad26.COV2.S versus sham groups within each challenge strain. *P < 0.05; **P < 0.01; ***P < 0.001.

We challenged all the immunized hamsters with 5 × 10^4^ TCID_50_ of either WA1/2020 or B.1.351 SARS-CoV-2 by the intranasal route. The sham immunization groups exhibited severe weight loss irrespective of the challenge strain (Fig. 4, A and B, and fig. S5), with mean peak weight loss of 17.6% for WA1/2020 and 14.4% for B.1.351. In contrast, hamsters vaccinated with Ad26.COV2.S and challenged with WA1/2020 SARS-CoV-2 exhibited a marked reduction in peak weight loss to a mean of 3.1%, similar to our previous results (*9*). Furthermore, hamsters immunized with Ad26.COV2.S and challenged with B.1.351 were robustly protected from weight loss, with a mean peak weight loss of 0.9%. These challenge outcomes were strongly correlated with prechallenge binding and neutralizing antibody titers, although the high degree of protection in Ad26.COV2.S-immunized animals limited potential to define a threshold of protection (fig. S6).

At day 14 after challenge, lung tissues were collected to analyze viral load. sgRNA and gRNA were detected in sham animals challenged with both WA1/2020 and B.1.351 variants at day 14 after challenge (Fig. 4, C and D). In the context of WA1/2020 strain challenge, a 1.5-log reduction in lung subgenomic viral load was observed in hamsters immunized with Ad26.COV2.S relative to the sham control group. Similarly, in the context of B.1.351 variant challenge, a 1.1-log reduction in subgenomic viral load was observed in Ad26.COV2.S-immunized animals relative to sham controls. In addition, at day 14 after challenge, serum was collected to analyze humoral responses, revealing increased binding and neutralizing antibody titers after exposure to either WA1/2020 or B.1.351 virus (fig. S7), supporting a role for immunologic control of infection and disease.

In parallel, lung tissue was analyzed at day 14 after challenge using a scoring rubric to quantify pathology and disease severity (table S1). We observed that vaccinated hamsters challenged with either WA1/2020 or B.1.351 had fewer pathological lesions compared with sham-vaccinated and challenged animals, resulting in statistically significant reductions in cumulative pathology score (WA1/2020, *P* < 0.01 and B.1.351, *P* < 0.05; fig. S8A). Ad26.COV2.S-vaccinated hamsters that were challenged with either WA1/2020 or B.1.351 exhibited preserved bronchiolar epithelial integrity and reduced pulmonary consolidation. Furthermore, only mild to moderate expansion of the interstitial spaces with inflammatory cells was observed, with minimal evidence of epithelial regeneration by day 14 after challenge, indicative of markedly reduced alveolar damage during peak inflammation as compared to sham-vaccinated and challenged animals (fig. S8, B to I).

## DISCUSSION

We (*9*, *13*) and others (*7*, *8*, *10*) have previously reported hamsters as a preclinical model for moderate to severe SARS-CoV-2. However, the impact of SARS-CoV-2 variants, including B.1.1.7 and B.1.351, on pathogenesis and clinical disease in hamsters remains to be fully defined. We demonstrate that challenge with these variants drives severe disease with similar magnitudes of weight loss. These findings are consistent with reported data revealing similar degrees of viral replication and disease pathology after challenge with variant strains including B.1.1.7 and B.1.351 (*19*). An important question in the context of human disease is whether circulating and emerging variants may evade natural immunity to SARS-CoV-2 infection and COVID-19 disease (*1*, *2*, *5*, *20*). We assessed whether infection with the WA1/2020 strain would provide protection against either homologous rechallenge or inoculation with variant strains. We observed robust protection against clinical disease and lung viral load for all rechallenge variants. This finding was observed in the context of reduced B.1.351-specific binding antibody titers elicited by primary WA1/2020 challenge, which could support further studies to define the threshold of protection for each variant strain.

In the context of Ad26.COV2.S vaccination, we observed reduced humoral immune responses in B.1.351-specific ELISA and ECLA binding assays, as well as pseudovirus neutralization assays (*3*, *6*). Despite reduced titers, similar protection against clinical evidence of disease was observed after challenge with either homologous WA1/2020 or heterologous B.1.351. These data suggest a role for cross-reactive binding and neutralizing antibodies to protect against disease after heterologous strain challenge. Future studies could seek to define thresholds of protection for B.1.351 or other circulating and emerging variants. In addition, future studies could explore the role of vaccine-elicited T cell responses in vaccine protection (*21*).

A limitation of the studies presented here is the relatively short time interval between initial exposure or immunization and challenge. Future durability studies could explore protection after an extended time interval. Furthermore, these studies primarily focused on clinical end point readouts, specifically body weight, given the unique potential of the hamster model for studying clinical disease. As such, all animals were followed for weight loss 14 days after challenge, and thus virologic analyses of lung tissues were only possible on day 14. Future studies could focus more directly on the potential for virologic control and transmission in hamsters or other animal models. For example, we have recently demonstrated the potential for immunity elicited by natural infection to drive strong yet partial restraint of heterologous strain replication in the nonhuman primate model (*22*).

Our results are consistent with previous reports demonstrating immunogenicity of Ad26.COV2.S in preclinical models and early clinical trials (*17*, *18*), as well as observed reduced cross-reactive humoral responses against B.1.351 in nonhuman primates. The protection from weight loss and pathological signs of disease in lungs in the context of heterologous challenge are also consistent with the observed protection in the phase 3 clinical trial of Ad26.COV2.S. Participants were protected from severe COVID-19 after Ad26.COV2.S vaccination, including in South Africa, where 95% of viruses sequenced were the B.1.351 variant (*14*). The consistency between clinical trials and preclinical models supports the use of the hamster model to study SARS-CoV-2 variants of concern in greater detail. Together, these findings suggest that immunity from natural infection or vaccination can provide robust short-term protection against heterologous strain challenge and support the extension of the hamster model to study emerging circulating variants of concern.

## MATERIALS AND METHODS

### Study design

Female and male golden Syrian hamsters (Envigo), 8 weeks of age, were randomly allocated to groups. For viral challenge or rechallenge, hamsters were administered SARS-CoV-2 in a volume of 100 μl (50 μl per nostril) by the intranasal route. After challenge, body weights were assessed daily. Body weight loss that exceeded 20% of the weight on the day of challenge was established as a humane endpoint euthanasia criteria. In vaccine studies, hamsters received a single immunization of 10^10^ viral particles of Ad26.COV2.S by the intramuscular route without adjuvant in 200 μl (100 μl per leg) of 5% sucrose dissolved in 1× phosphate-buffered saline (PBS). At indicated time points, peripheral blood was collected by the retro-orbital route to isolate serum for immunologic assays. Immunological and virological assays were performed blinded. All animal studies were conducted in compliance with all relevant local, state, and federal regulations and were approved by the BIOQUAL Institutional Animal Care and Use Committee.

### Variant strain SARS-CoV-2 viral stock generation and characterization

The WA1/2020 [USA-WA1/2020; Biodefense and Emerging Infections Research Resources Repository (BEI Resources), NR-5228] challenge stock was grown using a single passage in VeroE6 cells [American Type Culture Collection (ATCC)] and deep sequenced as described previously (*15*). The B.1.1.7 (USA/CA_CDC_5574/2020; BEI Resources, NR-54011) and B.1.351 (South Africa/KRISP-K005325/2020; BEI Resources, NR-54974) challenge stocks were prepared similar to as previously described (*15*) using a single passage in Calu-3 cells (ATCC HTB-55). Deep sequencing of these stocks revealed no mutations or deletions in the Spike protein greater than >2.5% frequency. TCID_50_ values for WA1/2020 and B.1.351 variants were quantified in VeroE6 cells.

### Enzyme-linked immunosorbent assay

RBD-specific binding antibodies were assessed by ELISA essentially as previously described (*15*, *16*). Briefly, plates were coated with SARS-CoV-2 RBD proteins (0.5 μg/ml; F. Krammer, Icahn School of Medicine at Mount Sinai), diluted in 1× PBS, and incubated at 4°C overnight. After incubation, plates were washed once with ELISA wash buffer (0.05% Tween 20 in 1× PBS) and blocked with 350 μl of casein (Thermo Fisher Scientific) per well. The block solution was discarded after 2 to 3 hours of incubation at room temperature, and plates were blotted dry. Threefold serial dilutions of hamster serum in casein block were added to wells, and plates were incubated for 1 hour at room temperature. Plates were then washed three times, and goat anti-hamster IgG horseradish peroxidase (SouthernBiotech), diluted in casein block, was added to wells and incubated at room temperature in the dark. After 1 hour, plates were washed three times, and 100 μl of SeraCare KPL 3,3′,5,5′-tetramethylbenzidine (TMB) SureBlue Start solution was added to each well. Development was halted with the addition of 100 μl of SeraCare KPL TMB Stop solution per well. The absorbance at 450 nm was recorded using a VersaMax microplate reader. The raw optical density (OD) values were transferred into GraphPad Prism for analysis. End point titers were interpolated using a sigmoidal four-parameter logistic fit to calculate the dilution at which the OD value would be equal to a value of 0.1.

### Electrochemiluminescence assay

ECLA plates (Meso Scale Discovery SARS-CoV-2 IgG cat. no.: N05CA-1; Panel 7) were designed and produced with up to nine antigen spots in each well, and assays were performed essentially as described previously (*23*). The antigens included WA1/2020, B.1.1.7, P.1, and B.1.351 spike and RBD proteins. The plates were blocked with 50 μl of Blocker A (1% bovine serum albumin in distilled water) solution for at least 30 min at room temperature shaking at 700 rpm with a digital microplate shaker. During blocking, the serum was diluted 1:5000 in Diluent 100 (Meso Scale Discovery). The plates were then washed three times with 150 μl of Wash buffer (0.5% Tween 20 in 1× PBS) and blotted dry, and 50 μl of the diluted samples were added in duplicate to the plates and set to shake at 700 rpm at room temperature for at least 2 hours. Secondary antibody was prepared using SouthernBiotech goat anti-hamster immunoglobulin G (IgG) detection antibody (cat. no.: 6060-01) conjugated to the Meso Scale Discovery (MSD) GOLD SULFO-TAG by NHS (*N*-hydroxysuccinimide)–ester chemistry per the manufacturer’s guidelines (cat. no.: R91AO-1). The plates were again washed three times, and 50 μl of tagged secondary antibody solution diluted 1:1000 in Diluent 100 was added to each well and incubated, shaking at 700 rpm at room temperature for at least 1 hour. Plates were then washed three times, and 150 μl of MSD GOLD Read buffer B was added to each well, and the plates were read immediately after on a MESO QuickPlex SQ 120 machine. MSD titers for each sample were reported as RLU, which were calculated as average sample RLU minus blank RLU for each sample. The limit of detection was defined as 1000 RLU for each assay.

### Pseudovirus neutralization assays

A SARS-CoV-2 pseudovirus expressing a luciferase reporter gene was generated in an approach similar to as described previously (*15*, *16*, *24*). The packaging construct psPAX2 (AIDS Resource and Reagent Program), luciferase reporter plasmid pLenti-CMV Puro-Luc (Addgene), and spike protein expressing pcDNA3.1-SARS CoV-2 S.dCT were cotransfected into human embryonic kidney (HEK) 293T (ATCC) cells using Lipofectamine 2000 (Thermo Fisher Scientific). To generate variant strain pseudoviruses for assays, pcDNA3.1-SARS-CoV-2 S.dCT specific to the sequences of WA1/2020, WA1/2020-D614G, B.1.1.7, or B.1.351 were used. After 48 hours, supernatant was collected, and pseudotype viruses were purified by filtration with a 0.45-μm filter. To determine the neutralization activity of the antisera from vaccinated animals, HEK293T target cells expressing human angiotensin converting enzyme 2 (hACE2) were seeded in 96-well tissue culture plates at a density of 1.75 × 10^4^ cells per well and incubated overnight. Dilutions of heat-inactivated serum were prepared and mixed with 50 μl of pseudovirus. This mixture was incubated at 37°C for 1 hour before adding to HEK293T-hACE2 cells. Forty-eight hours after infection, cells were lysed in Steady-Glo Luciferase (Promega) according to the manufacturer’s instructions. Neutralization titers were defined as the sample dilution at which a 50% reduction in RLUs (NT50) was observed relative to the average of control wells containing virus only.

### Genomic and subgenomic viral load assays

SARS-CoV-2 N gene gRNA and E gene sgRNA was assessed by reverse transcription polymerase chain reactions (RT-PCR) using primers and probes as previously described (*16*, *25*, *26*). Standards were generated by first synthesizing a gene fragment of the genomic N gene or subgenomic E gene (*26*). The gene fragments were subsequently cloned into a pcDNA3.1^+^ expression plasmid using restriction site cloning (Integrated DNA Technologies). The inserts were in vitro transcribed to RNA using the AmpliCap-Max T7 High Yield Message Maker Kit (CELLSCRIPT). Log dilutions of the standard were prepared for RT-PCR assays ranging from 1 × 10^10^ to 1 × 10^−1^ copies. Viral loads were quantified from lung tissue as follows: Total RNA was extracted on a QIAcube HT instrument using the RNeasy 96 QIAcube HT Kit according to the manufacturer’s specifications (QIAGEN). Standard dilutions and extracted total RNA from samples were reverse-transcribed using SuperScript VILO Master Mix (Invitrogen) according to the manufacturer’s specifications. A TaqMan custom gene expression assay (Thermo Fisher Scientific) was designed using the sequences targeting the E gene sgRNA (*26*). The sequences for the custom assay were as follows: forward primer, sgLeadCoV2.Fwd: CGATCTCTTGTAGATCTGTTCTC; E_Sarbeco_R: ATATTGCAGCAGTACGCACACA; and E_Sarbeco_P1 (probe): VIC-ACACTAGCCATCCTTACTGCGCTTCG-MGB. For the genomic N assays, the sequences for the forward (F) and reverse (R) primes and probe (P) were as follows: 2019-nCoV_N1-F: 5′-GACCCCAAAATCAGCGAAAT-3′; 2019-nCoV_N1-R: 5′-TCTGGTTACTGCCAGTTGAATCTG-3′; and 2019-nCoV_N1-P: 5′-FAM-ACCCCGCATTACGTTTGGTGGACC-BHQ1-3′.

Reactions were carried out in duplicate for samples and standards on the QuantStudio 6 and 7 Flex Real-Time PCR Systems (Applied Biosystems). The following thermal cycling conditions were used: initial denaturation at 95°C for 20 s, then 45 cycles of 95°C for 1 s, and 60°C for 20 s. Standard curves were used to calculate sgRNA copies, and copy number was normalized to the input weight of lung tissue (copies per gram); the quantitative assay sensitivity was 100 copies/g.

### Histopathology

Tissues were fixed in freshly prepared 4% paraformaldehyde for 24 hours, transferred to 70% ethanol, paraffin-embedded within 7 to 10 days, and block-sectioned at 5 μm. Slides were baked for 30 to 60 min at 65°C and then deparaffinized in xylene and rehydrated through a series of graded ethanol to distilled water. Slides were stained with hematoxylin followed by bluing using 0.25% ammonia water. Blinded assessment of tissue pathology was performed by a veterinary pathologist (A.J.M.).

### Statistical analyses

Analysis of immunologic, virologic, and body weight data was performed using GraphPad Prism 9.1.2 (GraphPad Software). Comparison of data between groups was performed using two-sided Mann-Whitney tests. Comparison of data between three or more groups was performed using Kruskal-Wallis tests with Dunn’s multiple comparisons test. Correlations were assessed by two-sided Spearmanʼs rank correlation tests. *P* values of less than 0.05 were considered significant.

## Notes

View/request a protocol for this paper from *Bio-protocol*.
