# Supplementary material for: Immunity elicited by natural infection or Ad26.COV2.S vaccination protects hamsters against SARS-CoV-2 variants of concern
Source: Sci Transl Med. 2021 Sep 7;13(618):eabj3789. doi: 10.1126/scitranslmed.abj3789 (PMC8818312; doi:10.1126/scitranslmed.abj3789)
Supplement: Supplementary file 1 — Figs. S1 to S8 Table S1 [file scitranslmed.abj3789_sm.pdf]

Science Translational Medicine
AAAS

Supplementary Materials for

Immunity elicited by natural infection or Ad26.COV2.S vaccination protects hamsters against SARS-CoV-2 variants of concern

Lisa H. Tostanoski *et al.*

Corresponding author: Dan H. Barouch, dbarouch@bidmc.harvard.edu

Sci. Transl. Med. **13**, eabj3789 (2021)
DOI: 10.1126/scitranslmed.abj3789

The PDF file includes:

Figs. S1 to S8
Table S1

Other Supplementary Material for this manuscript includes the following:

Data file S1

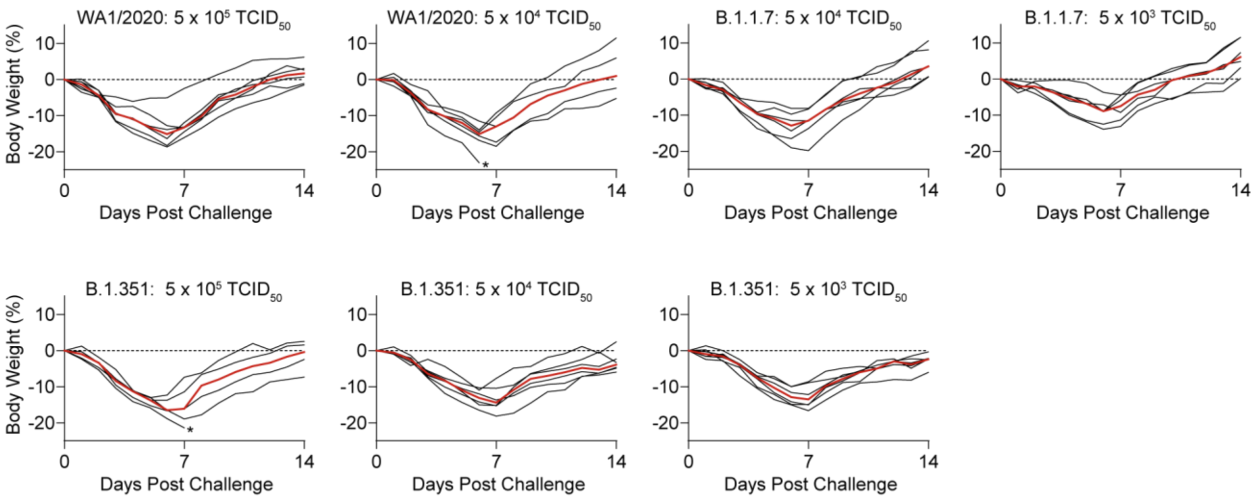

Fig. S1. SARS-CoV-2 challenge with variant strain viruses drives clinical disease in hamsters. Groups of Syrian golden hamsters were challenged intranasally with WA1/2020, B.1.1.7, or B.1.351 variant challenge virus, administered in 100μL of volume using the indicated dose of each challenge stock. Post-challenge, hamsters were monitored for fourteen days for clinical signs of disease. Weight traces of individual hamsters in each experimental group, with group medians indicated by the red lines. Asterisks (*) indicate animals that met euthanasia criteria of greater than 20% body weight loss. n=5-6 hamsters per group.

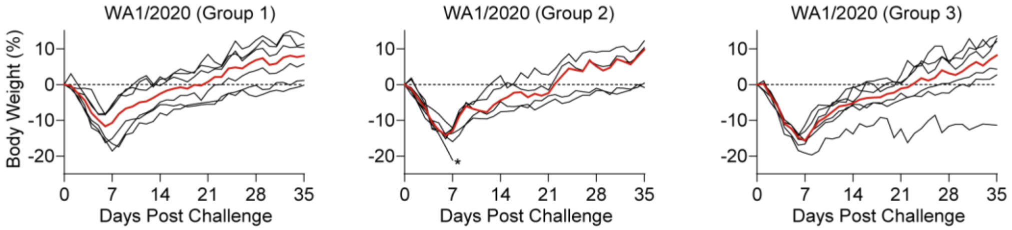

Fig. S2. Primary infection with WA1/2020 SARS-CoV-2 to investigate natural protection from homologous or heterologous re-challenge. Hamsters (n=18) were challenged on day 0 with 5 x 10^4 median tissue culture infectious dose ($TCID_{50}$) of WA1/2020 SARS-CoV-2 challenge stock by the intranasal route and monitored for 35 days post-challenge. To enable re-challenge with different variants at study day 35, hamsters were divided into three groups with similar weight loss distribution. Relative body weight in Groups 1 to 3 following primary challenge with WA1/2020 SARS-CoV-2 on day 0 is shown. Individual weight traces of hamsters are shown, with group medians indicated by the red lines. An asterisk (*) indicates an animal that met euthanasia criteria of greater than 20% body weight loss.

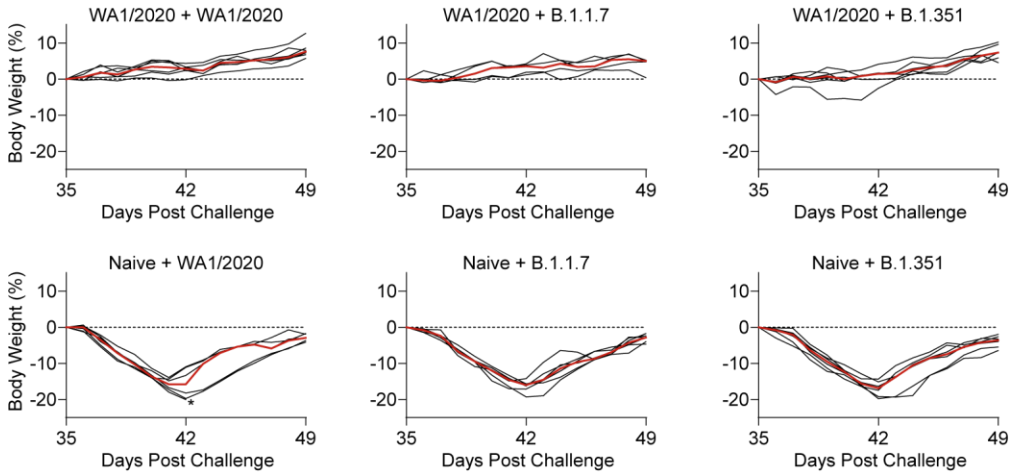

Fig. S3. Infection with WA1/2020 SARS-CoV-2 provides natural protection from homologous or heterologous strain re-challenge-induced weight loss. Three groups of hamsters (n=6 per group) were challenged on day 0 with 5 x 10^4 $TCID_{50}$ of WA1/2020 SARS-CoV-2 challenge stock by the intranasal route and monitored for 35 days post-challenge. On study day 35, groups were re-challenged with 5 x 10^4 $TCID_{50}$ of WA1/2020, B.1.1.7, or B.1.351 SARS-CoV-2. In addition, three groups of naïve, age-matched hamsters (n=6 per group) were challenged on study day 35 with the matched strains and doses. To allow comparison of weight loss following SARS-CoV-2 challenge or re-challenge on day 35, for the remainder of the study hamster weights were normalized to the weight on study day 35. Individual traces are displayed for hamsters in each experimental group from days 35-49, with group medians indicated in red lines.

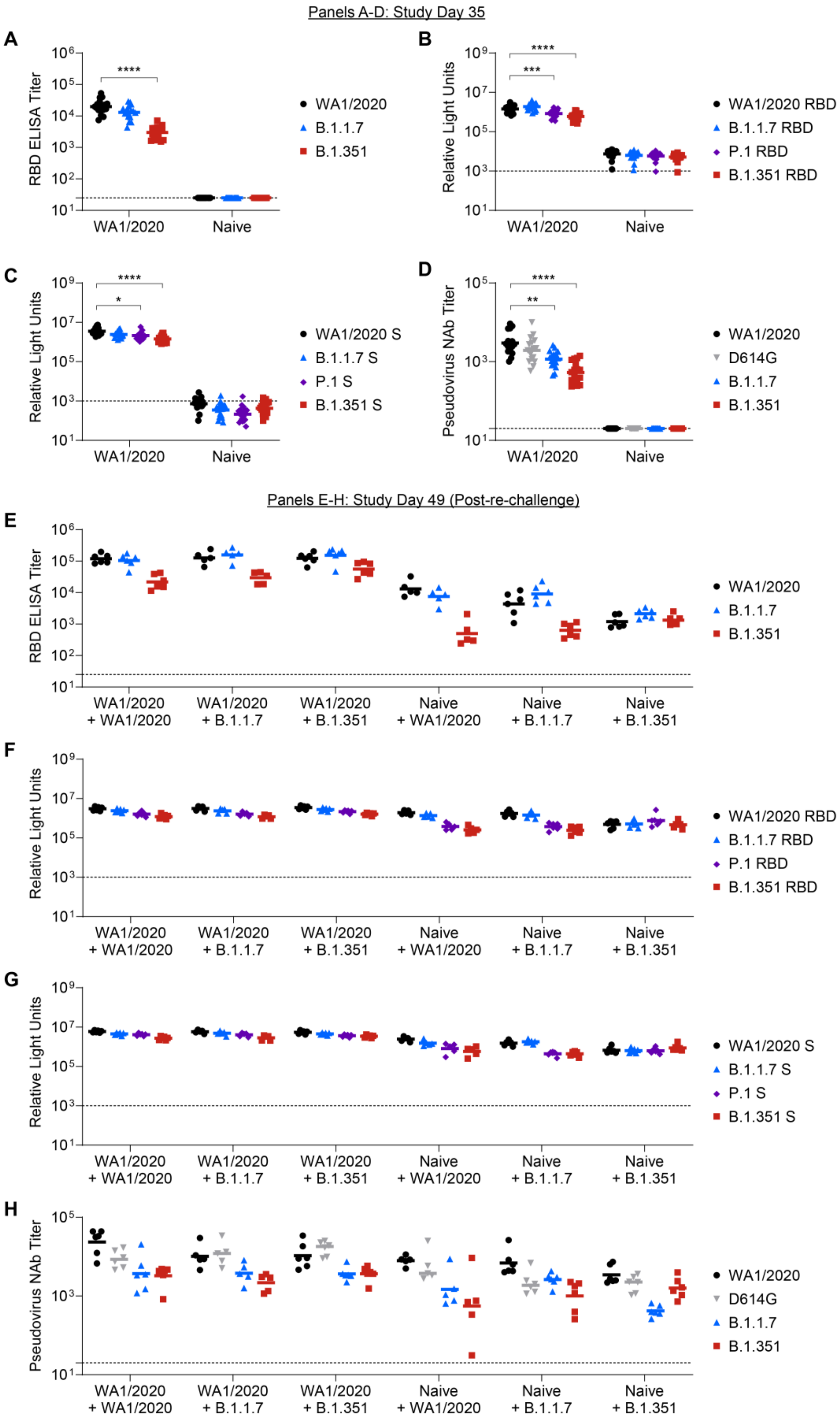

Panels A-D: Study Day 35
A
RBD ELISA Titer
WA1/2020
Naive
WA1/2020
B.1.1.7
B.1.351
B
Relative Light Units
WA1/2020 RBD
B.1.1.7 RBD
P.1 RBD
B.1.351 RBD
C
Relative Light Units
WA1/2020 S
B.1.1.7 S
P.1 S
B.1.351 S
D
Pseudovirus NAb Titer
WA1/2020
D614G
B.1.1.7
B.1.351
Panels E-H: Study Day 49 (Post-re-challenge)
E
RBD ELISA Titer
F
Relative Light Units
G
Relative Light Units
H
Pseudovirus NAb Titer
WA1/2020 + WA1/2020
WA1/2020 + B.1.1.7
WA1/2020 + B.1.351
Naive + WA1/2020
Naive + B.1.1.7
Naive + B.1.351

Fig. S4. Infection and re-challenge with SARS-CoV-2 homologous or heterologous strains elicits potent binding antibody responses. Hamsters (n=18) were challenged on day 0 with 5 x 10^4 $TCID_{50}$ of WA1/2020 SARS-CoV-2 challenge stock by the intranasal route and monitored for five weeks post-challenge. Serum was collected from all hamsters, as well as from additional naïve hamsters (n=18) to quantify binding and neutralizing antibody titers on study day 35. **(A)** Enzyme-linked immunosorbent assay (ELISA) titers representative of binding to WA1/2020, B.1.1.7, and B.1.351 RBD proteins are shown. **(B and C)** Electrochemiluminescence (ECLA) assays quantifying binding to WA1/2020, B.1.1.7, P.1, and B.1.351 RBD (B) and Spike (C) proteins. **(D)** Neutralizing titers specific to WA1/2020, B.1.1.7, and B.1.351 pseudoviruses are shown. Data indicate the 50% neutralization titers (NT50). Hamsters that had recovered from sWA1/2020 primary challenge were divided into three groups to be re-challenged with WA1/2020, B.1.1.7, or B.1.351 SARS-CoV-2 at 5 x 10^4 $TCID_{50}$. Similarly, the naïve hamsters were allocated into three groups to receive a primary challenge of the three variant strains to serve as internal positive controls. **(E)** On study day 49 (two weeks post-challenge or post-re-challenge), peripheral blood was again collected from all groups to quantify binding titers to WA1/2020, B.1.1.7, and B.1.351 RBD proteins by ELISA. **(F and G)** ECLA assays were conducted at study day 49 to quantify binding to variant RBD (F) and Spike (G) proteins. **(H)** Pseudovirus neutralizing antibody titers were quantified using the indicated variant strain pseudoviruses. In all panels, horizontal lines represent group geometric means. Dashed horizontal lines indicate the assay limits of quantitation

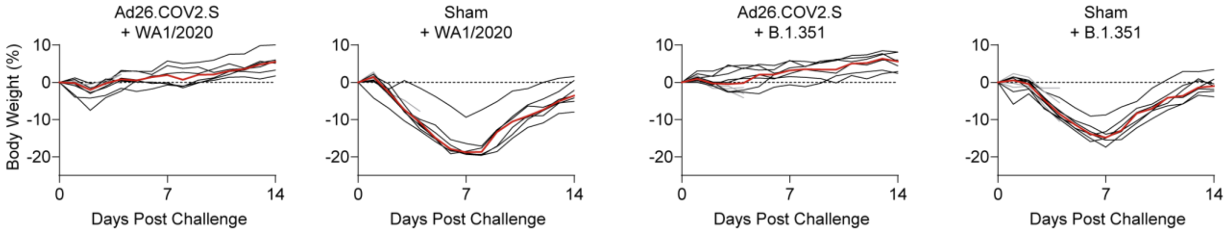

Fig. S5. Ad26.COV2.S protects hamsters from WA1/2020 or B.1.351 challenge-induced weight loss. Groups of hamsters (n=10 per group) were vaccinated with Ad26.COV2.S or sham, then challenged with 5 x 10^4 $TCID_{50}$ of either WA1/2020 or B.1.351 of SARS-CoV-2 by the intranasal route. Hamsters were monitored for body weight loss for 14 days post-challenge. Weight traces of individual hamsters in each experimental group are shown, with group medians indicated by red lines. Gray lines indicate animals with a planned early euthanasia at day 4 post-challenge (n=3 per group).

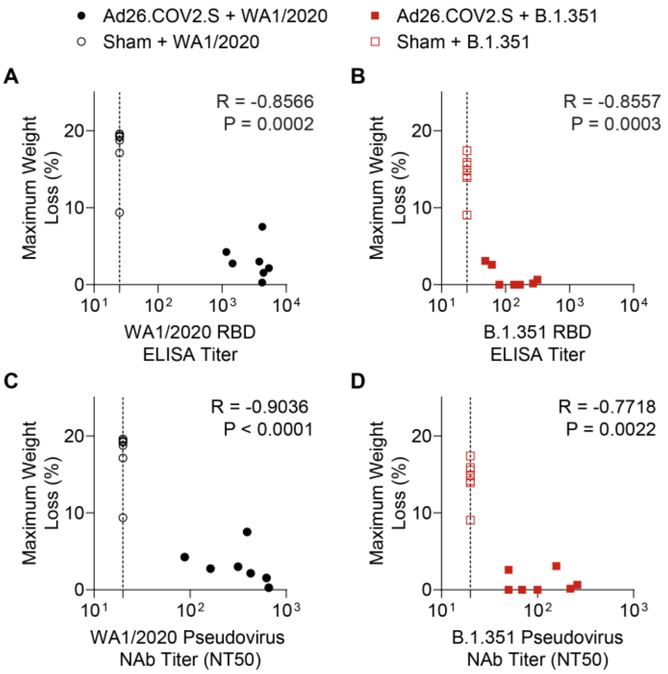

Fig. S6. Pre-challenge immune responses correlate with post-challenge weight loss. Groups of hamsters (n=10 per group) were vaccinated with either Ad26.CoV2. S or sham, then challenged with either WA1/2020 or B.1.351 SARS-CoV-2 by the intranasal route at week 5. (**A**) Correlation analysis of pre-challenge WA1/2020 RBD ELISA titers and post-challenge weight loss in the Ad26.COV2.S and sham groups challenged with WA1/2020 strain is shown. (**B**) Similar analysis of pre-challenge B.1.351 ELISA titer with post-B.1.351-challenge weight loss is shown. (**C and D**) Correlation analysis is shown for pre-challenge neutralizing antibody titers and post-challenge weight loss for WA1/2020 (C) and B.1.351 (D). In all panels, statistics indicate the result of a Spearman correlation analysis. Dashed vertical lines represent the assay limits of quantitation.

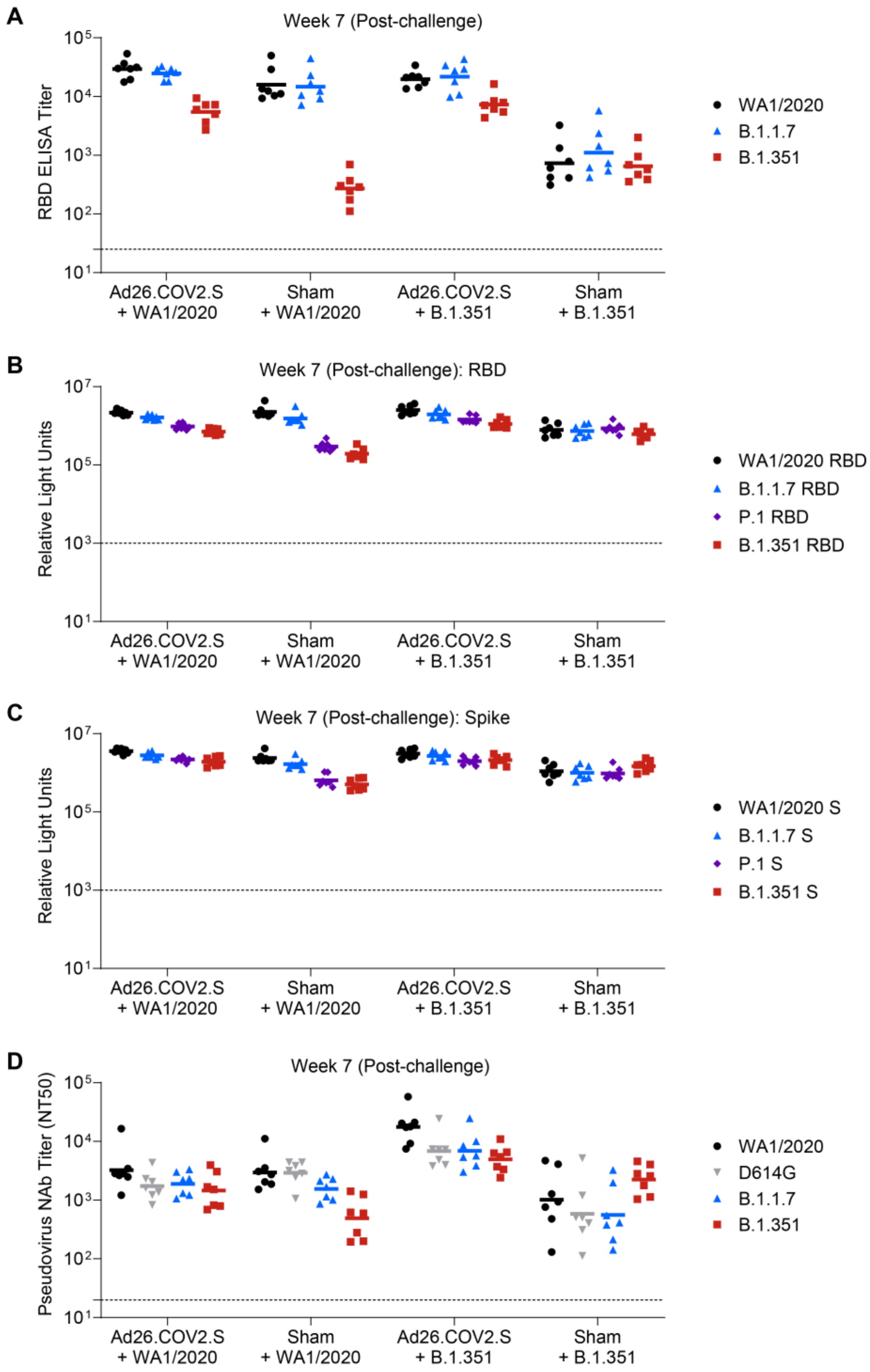

Fig. S7. Challenge with WA1/2020 or B.1.351 SARS-CoV-2 expands binding and neutralizing antibodies titers. Groups of hamsters (n=10 per group) were vaccinated with Ad26.COV2.S or sham (week 0), then challenged with either WA1/2020 or B.1.351 by the intranasal route (week 5). Fourteen days post-challenge (week 7), serum was collected to quantify humoral immune responses. (**A**) ELISA binding titers specific to the WA1/2020, B.1.1.7, and B.1.351 RBD proteins are shown. (**B and C**) Binding antibody assays were also performed using

the ECLA platform to quantify binding against variant RBD (B) and Spike (C) proteins. (**D**) Neutralizing antibody (NAb) titers against pseudoviruses expressing the WA1/2020, WA1/2020-D614G, B.1.1.7, and B.1.351 SARS-CoV-2 Spike are shown. Values displayed are the 50% neutralization titers (NT50). In all panels, horizontal lines indicate the group geometric means. Dashed horizontal lines indicate the assay limits of quantitation.

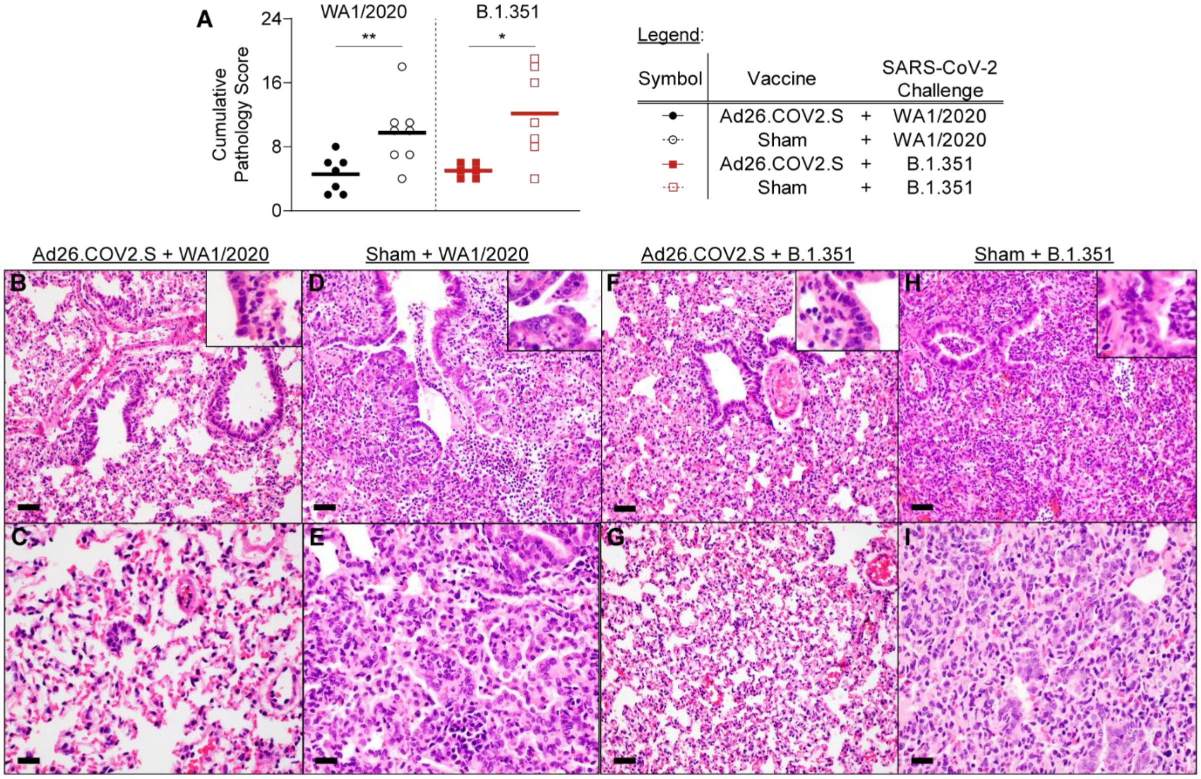

Fig. S8. Ad26.COV2.S restrains WA1/2020 or B.1.351 challenge-induced lung pathology. Groups of hamsters (n=10 per group) were vaccinated with Ad26.COV2.S or sham (week 0), then challenged with either WA1/2020 or B.1.351 by the intranasal route (week 5). Fourteen days post-challenge (week 7), lung tissue was collected for analysis. Lungs were insufflated and fixed with 10% neutral buffer formalin and processed for histopathology. Two lung lobes were evaluated per animal (n=6 per group). (**A**) Cumulative pathology scores per animal are shown (see table S1). Horizontal lines indicate group means. Statistics shown are the results of Mann Whitney tests, comparing Ad26.COV2.S versus sham groups within each challenge strain. * = $P < 0.05$, ** = $P < 0.01$. (**B to I**) Representative hematoxylin and eosin-stained images of bronchioles (insets), peribronchiolar parenchyma, and interstitium from Ad26.COV2.S-vaccinated (B, C, F, G) and sham vaccinated (D, E, H, I) hamsters 14 days following challenge with indicated strains of SARS CoV-2. Scale bars (B, D, F, H)= 50 μm; (C, E, G, I)= 20 μm.

Table S1. Regional Scoring Criteria for SARS CoV-2 pathology in hamsters.

**Bronchi/Bronchioles**	
**0**	No observed lesions.
**1**	Mild attenuation of the epithelium with multifocal loss of cilia
**2**	Moderate attenuation of the epithelium, sloughing of epithelium, loss of cilia, occasional syncytia formation; mild to moderate mixed inflammatory infiltrates
**3**	Marked attenuation of epithelium with diffuse loss of cilia, degeneration, necrosis and sloughing of epithelium; marked mixed inflammatory infiltrates
**Interstitium**	
**0**	No observed lesions.
**1**	Mild to moderate expansion of interstitium/consolidation with patchy distribution (affecting less than 50 % of parenchyma)
**2**	Mild to moderate expansion of interstitium/consolidation with diffuse distribution (greater than 50% of parenchyma), OR marked expansion of the interstitium/consolidation (affecting less than 50% of parenchyma)
**3**	Marked expansion of interstitium/consolidation with diffuse distribution (affecting greater than 50 % of parenchyma)
**Endothelial Changes/Endothelialitis**	
**0**	No observed lesions.
**1**	Mild to moderate reactive endothelium with rare subendothelial inflammation; occasional mild perivascular inflammatory infiltrates
**2**	Reactive endothelium with mild to moderate subendothelial mixed inflammation; mild to moderate perivascular mixed inflammatory infiltrates; occasional vascular wall inflammatory infiltrates
**3**	Marked and diffuse subendothelial mixed inflammation; marked and diffuse perivascular mixed inflammatory infiltrates; frequent vascular wall inflammation
**Alveolar spaces/syncytia**	
**0**	No observed lesions above background
**1**	Mild, focal to locally extensive alveolar filling with foamy macrophages and syncytia
**2**	Locally extensive alveolar filling with macrophages, viable and degenerative neutrophils
**3**	Severe neutrophilic infiltrates and necrosis with sheets of foamy macrophages and numerous alveolar epithelial syncytia
**Edema**	
**0**	Absent
**1**	Present, primarily perivascular
**2**	25 to 50% parenchyma with alveolar edema
**3**	Greater than 50% of parenchyma with alveolar edema
**Regeneration**	
**0**	None observed
**1**	Rare Type II pneumocyte hyperplasia
**2**	Multifocal ribbons of Type II pneumocyte hyperplasia with nuclear pleomorphism
**3**	Locally extensive Type II pneumocyte hyperplasia with nuclear pleomorphism, karyomegaly, occasional mitotic figures, and frequent piling up of nuclei
